# Detection of antibodies against *Ornithodoros moubata* salivary antigens and their association with detection of African swine fever virus in pigs slaughtered in central Uganda

**DOI:** 10.3389/fvets.2024.1328040

**Published:** 2024-03-28

**Authors:** Edrine B. Kayaga, Eddie M. Wampande, John E. Ekakoro, Rodney Okwasiimire, Aisha Nassali, Krista Ochoa, Cole Hauser, Dickson Ndoboli, Karyn A. Havas

**Affiliations:** ^1^Central Diagnostic Laboratory, College of Veterinary Medicine, Animal Resources, and Biosecurity, Makerere University, Kampala, Uganda; ^2^Department of Public and Ecosystem Health, College of Veterinary Medicine, Cornell University, Ithaca, NY, United States

**Keywords:** African swine fever virus, *Ornithodoros*, transmission, swine, Uganda

## Abstract

**Introduction:**

African swine fever (ASF) is an important disease of pigs in sub-Saharan Africa and Uganda and is threatening the pig population and agricultural economy of other continents. ASF virus (ASFV) can be transmitted from wild suids to domestic pigs through soft ticks of the *Ornithodoros* species. The aim of this study was to understand the relationship between domestic pigs’ *O. moubata* tick exposure and ASFV status.

**Methods:**

Pigs were sampled from six abattoirs in the Kampala metropolitan area of Uganda from May 2021 through June 2022. Blood, serum, and tissue samples were collected. Serum was tested for antibodies against the rtTSGP1 salivary antigens of *O. moubata* ticks using an indirect ELISA assay. Blood and tissue samples from pigs were tested to detect ASFV using qPCR. Probability of tick exposure was categorized based on sample-to-positive ratio cut-off points.

**Results:**

Out of 1,328 serum samples tested, there were 828 (62.3%) samples with a negligible probability; 369 (27.8%) with a medium probability; 90 (6.8%) with a high probability, and 41 (3.1%) with a very high probability of exposure to the *O. moubata* salivary antigen. There was a statistically significant association between the pigs’ *O. moubata* exposure and ASFV status with a higher proportion of pigs having a very high probability of infection if they were ASFV positive by blood, tonsil, and lymph nodes.

**Discussion:**

These results suggested that tick exposure was associated with ASFV transmission in Uganda. There were ASFV qPCR positive pigs that had no *O. moubata* exposure as well, which highlights that pig-to-pig and indirect contact transmission still play a significant role. This work highlights the need for further work in Uganda to investigate these transmission factors related to the *O. moubata* tick and ASFV transmission.

## 1 Introduction

The argasid or soft tick, *Ornithodorous moubata*, is a hematogenous ectoparasite and a vector of pathogens that transmits diseases in humans, poultry, and members of the suidae family (1). Geographically, this 3-host tick is common in sub-Saharan Africa with each developmental stage requiring a blood meal (2); each feeding allows for disease transmission. Further, ticks can be infected prior to hatching and feeding as transovarial transmission of pathogens can occur in infected ticks (3). Argasid ticks prefer living in sheltered environments such as cracks and fissures, the inside of animal burrows, pigsties, and human homesteads (4, 5). They take short meals on hosts when they are near the tick’s shelter and can survive for years in the absence of host as well (4). *O. moubata* is a vector for African swine fever virus (ASFV) (6, 7) as well as *Borrelia duttonii*, a pathogen that causes relapsing fever in humans (8).

ASFV is a large DNA arbovirus and is the only member of the *Asfarviridae* family (9). ASFV causes a febrile, hemorrhagic disease in domestic pigs and European wild boars that can have case fatality rates approaching 100% (10). Wart hogs in sub-Saharan Africa experience subclinical infections that contribute to maintenance of the virus in the sylvatic cycle (7, 11). There are 24 different genotypes classified based on sequencing of the p72 gene (12, 13). In Africa, ASFV is found across sub-Saharan Africa (14, 15) and was first reported in 1921 in Kenya (16). Today, it is recognized as endemic in Uganda as well (14). ASFV has negatively impacted the socio-economic well-being of pig farmers. For example, a study in Northern Uganda revealed that ASFV can lead to lost revenue and a path to more severe poverty amongst small holder pig farmers (17). Control of ASFV transmission is needed to reduce the deleterious effects of the virus on pig production.

It is necessary to understand the transmission dynamics of a disease in the target population to have a robust disease control program. ASFV has complex and diverse modes of transmission and maintenance (9). These include a sylvatic cycle and a domestic pig cycle (18). The sylvatic cycle includes the *O. moubata* soft tick as a vector of ASFV to wild pigs, particularly the warthog (19, 20). The tick is infected by feeding on a viremic warthog, once infected it can transmit the disease transovarially to its offspring and transtadially across its life stages remaining infective for up to 15 months (21, 22). Infected *O. moubata* ticks often transmit ASFV to naïve piglets (23). Infected warthogs can transmit disease through contact with domestic pigs, although this is not a significant cause of disease in domestic pigs. Further, domestic pigs can be infected when fed on by an infected soft tick (24). In the tick-domestic pig cycle, the virus is transmitted between infected ticks and naïve domestic pig when ticks are present in domestic pig habitats (9). Finally, there is the domestic cycle, which occurs in the absence of ticks where the virus is transmitted by direct or indirect contact from infected to non-infected domestic pigs (25, 26). All these pathways contribute to the persistence of the virus, and control strategies must consider each pathway since the different pathways require different resources and control methods (27).

Tick related ASFV transmission is difficult to assess and to manage. This is due to the long survival of the tick, the short feedings of the tick that prevent one from finding them on animals, and the difficulty in finding them without proper tools since they live in sheltered environments (4). An enzyme-linked immunosorbent assay (ELISA) was developed to detect if pigs had been bitten by the *O. moubata* tick (28). The *O. moubata* salivary proteins have properties that assist the tick in blood-sucking and facilitate transmission of pathogens to the host. These proteins block platelets activation and inhibit the complement cascade by preventing C5 activation into C5a which promotes the production of proinflammatory mediators (29, 30). This ELISA can be used to assess whether pigs have provided a blood meal for an *O. moubata* tick and can be used to assess the risk of exposure to this vector.

Numerous studies in Uganda have identified modes of transmission of ASFV between domestic pigs through direct and indirect pig-to-pig contact (31–34). However, the role of *O. moubata* ticks in the transmission of the ASFV to domestic pigs in Uganda has been inadequately studied, with only two studies, neither of which are in the peer-reviewed literature. A study in 1969 found 22% of warthogs in Queen Elizabeth National Park were seropositive for ASFV antibodies and 100% had *O. moubata* infected burrows, but none of the soft ticks were infected with ASFV (6). In 1994, it was reported that there was a high seroprevalence of ASFV antibodies in warthogs while only 0.017% of *O. moubata* ticks were infected with ASFV in Rwenzori National Park in western Uganda (20, 35). A more recent study from 2015 found that 10% of *O. moubata* ticks sampled were positive ASFV in two districts in Uganda and a 22% of ticks from two districts in Kenya were positive (36). Yet, there are limited studies that have evaluated the exposure of domestic pigs to ASFV through argasid ticks. Overall, there is a lack of research done in Uganda on the role of the *Ornithodoros* tick species in ASFV transmission. The purpose of this study was to describe the exposure of domestic pigs to the *O. moubata* tick and to compare pigs’ *O. moubata* exposure and ASFV status. This will allow for a further understanding of the role ticks may play in ASFV transmission in Uganda.

## 2 Materials and methods

### 2.1 Study area and design

A cross-sectional study was carried out at six pig abattoirs in the Kampala metropolitan area of Uganda from May 2021 through June 2022. The abattoirs were purposively selected as they receive the largest number of pigs from a wide geographic range. A stratified sampling plan was used and weighted based on annual number of pigs slaughtered at each abattoir. Systematic sampling of pigs occurred two to four days a month, with the days being randomly selected. This allowed for representative sampling of the pigs at the abattoirs. A total of 1,328 serum and blood samples were collected. The sample size was determined as part of a larger project where 100 ASFV positive samples were needed to fully characterize patterns in disease diagnostics and clinical presentations between infected pigs. Since the expected prevalence was 11.5% (37), over 1,200 pigs were needed to detect 100 ASFV positive pigs with 95% confidence and 5% error. Additional data for each pig were also collected and included farm size, district of origin, pig type (local breed, European breed, or mixed breed) and sex.

### 2.2 Sample collection and African swine fever virus testing

Each pig had blood, serum, tonsil, lymph nodes (submandibular, renal, and gastrohepatic), and spleen collected. Blood was collected from the jugular vein using 21-gauge needles into 10 mL vacutainer blood collection tube (Becton, Dickson and Company, Franklin Lakes, New Jersey, United States) for serum and EDTA tubes (Becton, Dickson and Company, Franklin Lakes, New Jersey, United States) for whole blood. In addition to blood and serum, lymph nodes (submandibular, renal, and gastro-hepatic), tonsils, and spleen samples were collected using separate gloves and instruments between each sample type from each pig after they were slaughtered. They were transported on ice to the Central Diagnostic Laboratory at Makerere University at the end of each sampling day.

The blood collection tubes were incubated overnight at 4°C to allow for maximum serum extraction. Serum was then separated from the blood clot through centrifugation at 1000 x g for 10 min (Eppendorf centrifuge 5,804, Germany) and stored at-20°C until tested using an ELISA. The whole blood and tissues were stored at-20°C until they were processed for DNA extraction. Blood and tissue preparation, DNA extraction, and qPCR testing for ASFV followed the US Department of Agriculture’s Foreign Animal Disease Diagnostic Laboratory protocols (38, 39). In brief, blood was diluted 1:1 with phosphate buffered saline (PBS) (Thermo Fisher Scientific, Waltham, Massachusetts, United States). As for tissues, 1 g of tissue was homogenized using the Stomacher 80 Biomaster (Seward Ltd., West Sussex, United Kingdom) and then combined with 9 mL of 1X PBS. This mixture was centrifuged at 1000 x g for 10 min and the supernatant was then collected for DNA extraction and subsequent testing on qPCR. Lymph nodes from each pig were pooled for analysis.

The DNA extraction and real-time PCR (qPCR) testing procedures for ASFV also followed the US Department of Agriculture’s Foreign Animal Disease Diagnostic Laboratory protocols as described previously (40). DNA was extracted using the Qiagen DNeasy blood and tissue kit (Qiagen, Hilden, Germany). The qPCR testing was done using a previously described assay (41) to detect ASFV nucleic acid. The TaqMan Fast Virus 1-Step Master Mix (Thermo Fisher Scientific, Waltham, Massachusetts, United States) along with the forward primer of 5’-CCTCGGCGAGCGCTTTATCAC-3′, reverse primer of 5’-GGAAACTCATTCACCAAATCCTT-3′, and probe of FAM-CGATGCAAGCTTTAT-MGB/NFQ (Eurofin Genomic, Munich, Germany) were used along with the VetMax Xeno DNA internal positive control (IPC) (Thermo Fisher Scientific, Waltham, Massachusetts, United States) and the VetMax Xeno IPC LIZ Assay (Thermo Fisher Scientific, Waltham, Massachusetts, United States).

Not all samples were tested for ASFV due to a stop in work required by the funding entity on all projects in Uganda in June 2023. In total, 1316 blood samples were tested, 1254 spleen samples, 1208 lymph nodes, and 1247 tonsils. The samples tested varied by pig, but all serum samples were tested, and every pig had at least one other sample type tested.

### 2.3 Testing for *Ornithodoros moubata* exposure in swine sera

An indirect ELISA for detection of antibodies in swine sera against the *O. moubata* saliva lipocalin recombinant truncated tick salivary gland protein 1 (rTSGP1) antigen in pig serum developed at the Superior Council of Scientific Investigations in the Institute of Natural Resources and Agrobiology of Salamanca (CSIC, IRNASA, Salamanca, Spain) was used (42). The procedure was as follows. First, the polystyrene 96-well plates (Fisher Scientific, United States), were coated by adding 100 ng of *O. moubata* rtTSGP1 antigen (CSIC IRNASA, Salamanca, Spain) in 0.5 M bicarbonate buffer (Sigma, United States) with a pH of 9.6 to each well. Plates were incubated overnight at 4°C to allow the antigen to attach to the plate. Plates were washed five times with 200 μL/well 0.05% Tween 20 (Thermo Scientific, United States) in phosphate buffered saline (PBS) with a pH of 7.2. Immediately, 200 μL/well of 1% bovine serum albumin in PBS (Sigma, United States) was added and the plate incubated for 1 h at 37°C to block non-specific binding sites. Plates were again washed five times with 200 μL/well of 0.05% Tween 20 in PBS (TPBS) to remove the non-specific binding molecules. Next, 100 μL/well of diluted (1/300) sera and controls in 0.05% TPBS were added to each well. The plate was then incubated at 37°C for 1 h to allow the anti-rTSGP1 antibodies in the test sera to bind to the rTSGP1 antigen coated on the plates. After another wash, peroxidase-labelled anti-pig IgG (Sigma, United States) was added at 1/10,000 dilution in TPBS and the plates incubated further for 1 h at 37°C. After a final washing step, the reaction was developed in the dark using 100 μL/well of orthophenylene diamine substrate (Sigma, United States). The reaction was stopped with 100 μL/well of 3 N sulfuric acid (Wagtech Intl. ltd., UK) and the plates were read at 492 nm in an ELISA reader (Thermo Scientific Multiscan FC). The optical density of serum (OD_s_) for the negative (NC) and positive controls (PC) were 0.2 and 1.0, respectively. The serological index (SI) for each well’s optical density was calculated using the following formula (42):

$$\left[\left(NC-ODs\right)/\left(NC-PC\right)\right]\times 100$$

The SI was used to calculate the sample to positive (SP) ratio and samples were classified as negative (negligible probability of tick exposure) (SP < 0.10), weak positive (medium probability) (0.11 ≥ SP ≤ 0.30), positive (high probability) (0.31 ≥ SP ≤ 0.50), and strong positive (very high probability) (SP > 0.50) based on previously published work (43, 44). Interpretation was done as per the kit directions and previous publications that developed cut-off points based on the false positive rate or specificity of the assay at different SP ratios (43, 44).

### 2.4 Data analysis

#### 2.4.1 Summary and inferential statistics

Percent seropositivity and frequency were calculated for each probability of exposure to the *O. moubata* tick and stratified by the ASFV infection status by blood and tissue type based on the results from the qPCR testing from the same pig when data was available for both sample type. The association between *O. moubata* exposure status and overall ASFV infection status was evaluated using a Pearson chi-squared test as well as to measure the association to the sex of the pig. Multiple comparisons were done when evaluating antibody levels to pig type through use of a Bonferroni adjustment to the level of significance. Microsoft Excel v16.70 (Microsoft Corporation, Redmond, Washington, United States) and STATA version 16.1 IC (Stata Corp, College Station, Texas, United States) were used.

#### 2.4.2 Descriptive spatial summary

For purposes of mapping, serology results from pigs with an unknown district origin were excluded from the analysis (57/1328, 4.3%). All medium, high, and very high probability *O. moubata* exposure results were re-categorized as positive. This created a binary outcome of negative and positive results that could be summarized as a seroprevalence at the district of pig origin level. No districts were excluded due to small sample sizes. The seroprevalence was calculated using a Proc Freq procedure in SAS (version 9.4, SAS Institute Inc., Cary, NC). District level geographic information system (GIS) data for Uganda were downloaded from the United Nations’ High Commissioner for Refugee Operational Data Portal (https://data.unhcr.org/en/documents/details/83043; Accessed March 30, 2023). A choropleth map of the seroprevalence of *O. moubata* exposure among pigs was created using QGIS Firenze version 3.28.1.^1^

## 3 Results

### 3.1 Serologic results for *Ornithodoros moubata* exposure

Of the 1,328 serum samples tested, 828 (62.35%) tested negative (negligible probability of exposure based on S/*p* values) and 500 (37.7%) tested positive for some level of *O. moubata* salivary antigen exposure. The probability of *O. moubata* exposure varied. There were 369 (27.8%) weak positive (medium probability of exposure) samples, 90 (6.8%) positive (high probability) samples, and 41 (3.1%) strong positive (very high probability) samples (see Table 1).

**Table 1:** Summary of pigs’ *Ornithodoros moubata* salivary antigen exposure status stratified across sex and pig type for pigs sampled at Kampala metropolitan abattoirs from May 2021 through June 2022.

	*O. moubata* ELISA result								
	Negative		Weak positive		Positive		Strong positive		*p* value*
*N* = 1,328	**#**	**%**	**#**	**%**	**#**	**%**	**#**	**%**	
**Samples**	828	62.35	369	27.8	90	6.8	41	3.1	
**Pig sex**	#	%	#	%	#	%	#	%	0.136
Male, *n* = 593	388	65.4	149	25.1	36	6.1	20	3.4	
Female, *n* = 728	435	59.75	218	29.9	54	7.4	21	2.9	
Unknown, *n* = 7	5	71.4	2	28.6	0	0.0	0	0.0	
**Pig type**	**#**	**%**	**#**	**%**	**#**	**%**	**#**	**%**	<0.001
Local breed, *n* = 199	91	45.7	78	39.2	18	9.0	12	6.0	a**, b
European breed, *n* = 757	501	66.2	194	25.6	38	5.0	24	3.2	a
Cross, *n* = 351	224	63.8	90	25.6	32	9.1	5	1.4	b
Unknown, *n* = 21	12	57.1	7	33.3	2	9.5	0	0.0	

*Chi-squared analysis excluded unknown findings and just tested results of animals with known classifications to reduce any misclassification bias. ** Lower case letters indicate the pairs that were significantly different from one another when the omnibus chi-squared was significand pair-wise comparisons were done using a Bonferroni adjusted level of significance.

Table 1 summarizes tick exposure based on pig characteristics. Significant associations were found between *O. moubata* exposure status and pig type (*p*-value <0.001). Based on pair-wise comparisons, there were differences in *O. moubata* exposure between local pigs and European breeds and local pigs and cross bred pigs, but no differences between European pigs and cross-bred pigs. Local breed pig serum samples had the lowest seronegativity (45.7%; 91/199) of all pig types. Within the local breed pig group, local breed had the highest seropositive in the weak positive and strong positive ELISA result. There was no statistically significant association between ELISA result and a pig’s sex. As for the ASFV status of pigs in this study, based on the combined results from blood and tissues 65.8% (794/1206) were positive for ASFV. The tonsils had the greatest percent positivity (474/1246, 38.0%), followed by spleen (395/1254, 31.5%), lymph nodes (453/1207, 37.5%), and then blood (201/1315, 15.3%) (see Table 2).

**Table 2:** Summary of pigs’ *Ornithodoros moubata* salivary antigen exposure status stratified across the African swine fever real-time PCR assay by sample type.

	*O. moubata* exposure status								
*N = 1,328*	Negative		Weak positive		Positive		Strong positive		*p*-value
Samples	828	62.35	369	27.8	90	6.8	41	3.1	
***ASFV status***									
**Blood**	#	%	#	%	#	%	#	%	0.002
Positive, *n* = 201	108	53.7	60	29.85	20	9.95	13	6.5	
Negative, *n* = 1,114	710	63.7	306	27.5	70	6.3	28	2.5	
Unknown^*^, *n* = 13	10	76.9	3	23.1	0	0.0	0	0.0	
**Spleen**									0.348
Positive, *n* = 395	241	61.0	117	29.6	22	5.6	15	3.8	
Negative, *n* = 858	541	63.05	229	26.7	64	7.5	24	2.8	
Unknown^*^, *n* = 75	46	61.3	23	30.7	4	5.3	2	2.7	
**Lymph nodes**									0.012
Positive, *n* = 453	265	58.5	129	28.5	39	8.6	20	4.4	
Negative, *n* = 754	492	65.3	204	27.1	41	5.4	17	2.25	
Unknown^*^, *n* = 121	71	58.7	36	29.75	10	8.3	4	3.3	
**Tonsil**									0.026
Positive, *n* = 474	282	59.5	133	28.1	37	7.8	22	4.6	
Negative, *n* = 772	500	64.8	210	27.2	46	6.0	16	2.1	
Unknown^*^, *n* = 82	46	56.1	26	31.7	7	8.5	3	3.7	
**Overall**									0.363
Positive, *n* = 794	479	60.3	224	28.2	61	7.7	30	3.8	
Negative, *n* = 412	268	65.0	113	27.4	23	5.6	8	1.9	
Unknown^*^, *n* = 122	81	66.4	32	26.2	6	4.9	3	2.5	

*Chi-squared analysis excluded unknown findings and just tested results of animals with known classifications to reduce any misclassification bias. “N” indicated the total number of samples tested for *O. moubata*. If any of the tissue ASFV qPCR results were missing for any pig they were classified as unknown in the Overall category.

Comparison of the ASFV status based on qPCR results with the *O. moubata* exposure based on serology for each pig was summarized in Table 2. *O. moubata* exposure status was significantly associated with the blood samples’ (*p*-value = 0.002), lymph nodes’ (*p*-value = 0.012), and tonsils’ (*p*-value = 0.026) ASFV status. Among the pigs with positive blood samples for ASFV, 6.5% (13/201) also had strong positive ELISA results, while the pigs with blood samples negative for ASFV had a 2.5% (28/1114) seropositivity on the ELISA. In addition, 53.7% (108/201) of pigs that tested positive for ASFV tested negative for *O. moubata* antibodies and 63.7% (710/1114) of the ASFV negative blood samples were also negative for *O. moubata* exposure. A similar trend was seen with tonsils and lymph nodes as with blood. Furthermore, ASFV positive pigs had a higher seroprevalence for the category of very high probability of exposure (strong positive) than ASFV negative pigs. The seroprevalence was 2.6 times (6.5%/2.5%) higher in pigs whose blood was ASFV positive (*p*-value = 0.01), 2.0 times higher (4.4%/2.25%) in pigs whose lymph nodes were positive (*p*-value = 0.069), and 2.2 times higher (4.6%/2.1%) in pigs whose tonsils were positive (*p*-value = 0.099). The qPCR results on the spleen did not detect any association between the ASFV status and the pig’s probability of exposure to the *O. moubata* tick, nor was there an association when considering the pigs overall ASFV status (based on any of the blood or tissues samples being positive for ASFV nucleic acid).

### 3.2 Mapping of exposure to *Ornithodoros moubata*

**Figure 1:**
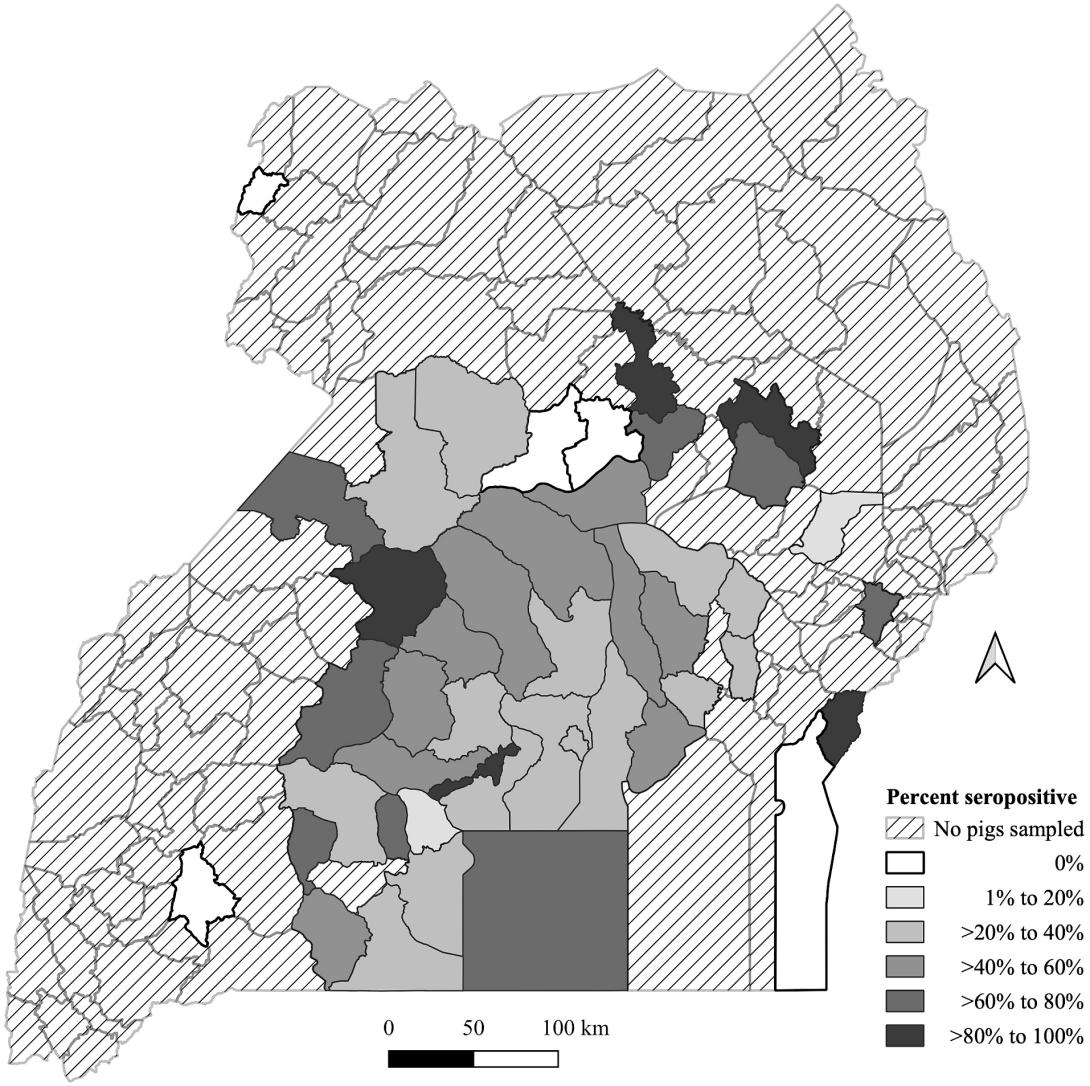
Geographic distribution of *Ornithodoros moubata* exposure based on antibody seroprevalence in pigs by the Ugandan district of origin from pigs sampled at Kampala metropolitan abattoirs from May 2021 through June 2022. A map depicted Ugandan districts in a color-coded in grey scale to describe the proportion of pigs with tick exposure.

*O. moubata* exposure seroprevalence is summarized in Figure 1. Data were available from 45 (33.1%) districts across Uganda out of 136 districts in the map. Pig origins were primarily from the central region of Uganda and were minimal outside of that region. Only five districts had pigs with no exposure to the *O. moubata* tick, of these two had a shared border in the north central area. The median number of pigs per district was 9 with a minimum of 1 and a maximum of 361. There were 25 districts with less than 10 pigs (Supplementary Table S1). There were five (3.7%) districts that had >80% seroprevalence and none of these districts had a common border with one another. Eight districts (5.9%) had >60 to 80% seropositivity, 10 (7.4%) had >40 to 60% seropositivity, 15 (11.0%) had >20 to 40% seropositivity, and two (1.5%) had >0 to 20% seropositivity for *O. moubata* tick exposure.

## 4 Discussion

There was minimal work in the literature regarding the role of the *O. moubata* tick in ASFV transmission in Uganda. To the best of our knowledge this is the first study to document the exposure of domestic pigs to the *O. moubata* tick. We found that 37.7% (500/1327) of pigs had some probability of exposure to the tick. There was a statistically significant association between the probability of tick exposure and ASFV status based on blood (*p*-value = 0.002), lymph nodes (*p*-value = 0.012), and tonsils (*p*-value = 0.026) samples. There was no association between spleen samples (*p*-value = 0.348) ASFV status or the overall ASFV status of the pig (*p*-value = 0.313) and the *O. moubata* exposure. It is unclear why different tissues have different relationships with *O. moubata* exposure. Understanding this will require further research that could include pathogenesis studies of infection through the *O. moubata* tick. These results show evidence of exposure to domestic pigs raised for slaughter and suggest a relationship between the *O. moubata* exposure status and the ASFV detection status of these pigs.

Further work will help identify where and how tick control programs need to be implemented and where monitoring is needed. A study out of South Africa showed there were ASFV seropositive warthogs in an area thought to be free of disease and further investigation revealed that the *Ornithodoros* spp. were also infected with ASFV in that area (45). This suggests that the area was initially declared free based on the pig status but not based on the *Ornithodoros* spp. ASFV status. Without a baseline understanding of the range of infection in warthogs and ticks, ongoing monitoring, control programs cannot be efficient. Uganda currently lacks a comprehensive baseline understanding of where the *O. moubata* tick is and whether it is infected with ASF. This knowledge is needed to know whether this soft tick contributes to the sylvatic and domestic cycle of pig infection.

This study shows that there is tick-exposure throughout the central region of Uganda but was not able to determine interactions between domestic pigs and the *O. moubata* tick in other areas. Therefore, it is not clear if this association between tick exposure and ASFV status is found throughout Uganda as our sampling only represented pigs from 45 out of 136 districts. There are only a handful of additional studies showing any evidence of tick exposure. One study conducted along the eastern Uganda-Kenya border determined that the seroprevalence of tick exposure for the Ugandan district of Tororo was 5 and 15% from the Ugandan district of Busia (36); our study did not have any pigs that originated from Tororo but 2 out of 3 pigs sampled from Busia were seropositive. Another study found a very low infectivity rate in ticks of 0.017% in Rwenzori National Park (11). Rwenzori National Park is in the western border of Uganda towards the south, and none of the pigs in this study originated from districts near this park. Further studies at the district level are needed to summarize the tick exposure more accurately. This study summarized findings from pigs sent to Kampala for slaughter, which may not be representative of the pig herd in all districts.

This study looked at pigs with antibodies against *O. moubata*, but *O. porcinus porcinus* is also present in Uganda (46) and *O. savignyi* is also a competent vector and is found in Africa (47). The salivary protein used to detect antibodies in the ELISA used in this study shows homology between *O. moubata* and *O. savignyi* but there was no information regarding its relationship to *O. porcinus porcinus* (28). So, detection of tick exposure may be limited and depending upon which tick is most common, could have a significant impact on our ability to detect exposure. Nonetheless, clearly there was *O. moubata* exposure and an association between a pig’s ASFV and *O. moubata* exposure status.

This work also generated further hypotheses. The highest tick exposure level was in the local breed pigs and this was similar to results reported from a study along the Uganda-Kenya border (36). Since the ELISA measures exposure to the tick, these differences may have more to do with housing, if there are differences in housing among pig breed types, or potentially a preference by the tick for the local breed. Different housing methods are used in Uganda in the areas these pigs were sourced from, including containment, free-roaming, and tethering of pigs or a mix of different methods. Yet, associations with different breeds were not determined as multiple farms had various housing methods and different breeds (48). In the present study, it was impossible to collect accurate and reliable information from pig traders at the abattoirs on pig housing at the source farms. Further work is needed to determine if specific breeds are associated with specific housing and if the housing method carries different risks of *O. moubata* exposure.

This study corroborates the role of the *O. moubata* tick in ASFV transmission in Uganda and should be a focus of disease control efforts. It is the first step in understanding the role of the tick vector in ASFV transmission in the country and showed that exposure occurs across all regions and through multiple districts. Further work is needed to elucidate the major factors associated with *O. moubata* exposure in Uganda, the distribution of *O. moubata* infected with ASFV, and methods to limit transmission to domestic swine.

## Data availability statement

The data used and analyzed for this study are available from the corresponding author upon reasonable request. Requests to access the datasets should be directed to KH, kah47@cornell.edu.

## Ethics statement

The studies involving humans were approved by Cornell University’s Institutional Review Board Human Research Protection Office of the US Army Medical Research and Development Command College of Veterinary Medicine, Animal Resources, and Biosecurity, Makerere University Higher Degrees Research Committee Uganda National Council for Science and Technology. The studies were conducted in accordance with the local legislation and institutional requirements. Written informed consent for participation was not required from the participants or the participants’ legal guardians/next of kin because the research was determined to be exempt from human research since none of the questions pertained to people and only to the pigs. The animal studies were approved by Cornell University’s Institutional Animal Care and Use Committee US Army Medical Research and Development Command’s Animal Care and Use Review Office College of Veterinary Medicine, Animal Resources, and Biosecurity, Makerere University Higher Degrees Research Committee Uganda National Council for Science and Technology. The studies were conducted in accordance with the local legislation and institutional requirements. Written informed consent was not obtained from the owners for the participation of their animals in this study because verbal consent was obtained from each pig trader who owned the pig as they were interviewed on pig origin information and reimbursed 20000 Ugandan shilling for their time. Each data sheet filled out is paired with a set of samples and each data sheet reflects a verbal consent.

## Author contributions

EK: Data curation, Formal analysis, Investigation, Methodology, Resources, Validation, Writing – original draft, Writing – review & editing. EW: Conceptualization, Funding acquisition, Methodology, Project administration, Supervision, Validation, Writing – review & editing. JE: Conceptualization, Data curation, Formal analysis, Investigation, Methodology, Visualization, Writing – review & editing. RO: Data curation, Investigation, Methodology, Writing – review & editing. AN: Data curation, Investigation, Writing – review & editing. KO: Data curation, Investigation, Writing – review & editing. CH: Data curation, Investigation, Writing – review & editing. DN: Conceptualization, Funding acquisition, Project administration, Resources, Writing – review & editing. KH: Conceptualization, Formal analysis, Funding acquisition, Methodology, Project administration, Resources, Supervision, Visualization, Writing – original draft, Writing – review & editing.

## Footnotes

^1^ qgis.org
