# Supplementary material for: Detection of antibodies against Ornithodoros moubata salivary antigens and their association with detection of African swine fever virus in pigs slaughtered in central Uganda
Source: Front Vet Sci. 2024 Mar 28;11:1328040. doi: 10.3389/fvets.2024.1328040 (PMC11007201; doi:10.3389/fvets.2024.1328040)
Supplement: Supplementary file 1 [file Table_1.docx]

Supplementary Material

Detection of antibodies against *Ornithodoros moubata* salivary antigens and their association with detection of African swine fever virus in pigs slaughtered in central Uganda.

# Supplementary Table 1: Summary statistics of *Ornithodoros moubata* exposure seroprevalence and pig sample sizes of pigs sampled from Kampala metropolitan area abattoirs based on their administrative district of origin.

|  | **Seronegative *O. moubata* exposure** | | **Seropositive *O. moubata* exposure** | | **Total Pigs from District** |
| --- | --- | --- | --- | --- | --- |
| **District** | **#** | **%** | **#** | **%** | **#** |
| Amolatar | 15 | 46.9 | 17 | 53.1 | 32 |
| Amuria | 0 | 0 | 1 | 100 | 1 |
| Apac | 3 | 100 | 0 | 0 | 3 |
| Buikwe | 3 | 50 | 3 | 50 | 6 |
| Bukomansimbi | 6 | 37.5 | 10 | 62.5 | 16 |
| Busia | 0 | 0 | 2 | 100 | 2 |
| Butambala | 0 | 0 | 1 | 100 | 1 |
| Buyende | 4 | 66.7 | 2 | 33.3 | 6 |
| Dokolo | 2 | 33.3 | 4 | 66.7 | 6 |
| Gomba | 9 | 50 | 9 | 50 | 18 |
| Hoima | 1 | 25 | 3 | 75 | 4 |
| Iganga | 8 | 72.7 | 3 | 27.3 | 11 |
| Jinja | 8 | 72.7 | 3 | 27.3 | 11 |
| Kalangala | 1 | 20 | 4 | 80 | 5 |
| Kaliro | 6 | 66.7 | 3 | 33.3 | 9 |
| Kalungu | 7 | 87.5 | 1 | 12.5 | 8 |
| Kampala | 9 | 60 | 6 | 40 | 15 |
| Kamuli | 22 | 45.8 | 26 | 54.2 | 48 |
| Kassanda | 1 | 50 | 1 | 50 | 2 |
| Kayunga | 34 | 58.6 | 24 | 41.4 | 58 |
| Kiboga | 12 | 48 | 13 | 52 | 25 |
| Kiryandongo | 2 | 66.7 | 1 | 33.3 | 3 |
| Kumi | 6 | 85.7 | 1 | 14.3 | 7 |
| Kwania | 1 | 100 | 0 | 0 | 1 |
| Kyankwanzi | 0 | 0 | 8 | 100 | 8 |
| Kyotera | 17 | 68 | 8 | 32 | 25 |
| Lira | 0 | 0 | 5 | 100 | 5 |
| Luweero | 79 | 73.8 | 28 | 26.2 | 107 |
| Lyantonde | 1 | 20 | 4 | 80 | 5 |
| Maracha | 1 | 100 | 0 | 0 | 1 |
| Masaka | 126 | 68.5 | 58 | 31.5 | 184 |
| Masindi | 7 | 77.8 | 2 | 22.2 | 9 |
|  | **Seronegative *O. moubata* exposure** | | **Seropositive *O. moubata* exposure** | | **Total Pigs from District** |
| **District** | **#** | **%** | **#** | **%** | **#** |
| Mbale | 1 | 33.3 | 2 | 66.7 | 3 |
| Mbarara | 2 | 100 | 0 | 0 | 2 |
| Mityana | 9 | 75 | 3 | 25 | 12 |
| Mpigi | 46 | 74.2 | 16 | 25.8 | 62 |
| Mubende | 4 | 28.6 | 10 | 71.4 | 14 |
| Mukono | 50 | 66.7 | 25 | 33.3 | 75 |
| Nakaseke | 17 | 53.1 | 15 | 46.9 | 32 |
| Nakasongola | 8 | 47.1 | 9 | 52.9 | 17 |
| Namayingo | 1 | 100 | 0 | 0 | 1 |
| Rakai | 4 | 44.4 | 5 | 55.6 | 9 |
| Soroti | 4 | 23.5 | 13 | 76.5 | 17 |
| Ssembabule | 16 | 61.5 | 10 | 38.5 | 26 |
| Wakiso | 241 | 67.1 | 118 | 32.9 | 359 |
| Median | 6 | 58.6 | 4 | 41.4 | 9 |
| Minimum | 0 | 0 | 0 | 0 | 1 |
| Maximum | 241 | 100 | 118 | 100 | 359 |
| 25th Percentile | 1 | 33.3 | 2 | 27.3 | 4 |
| 75th Percentile | 12 | 72.7 | 10 | 66.7 | 25 |

**
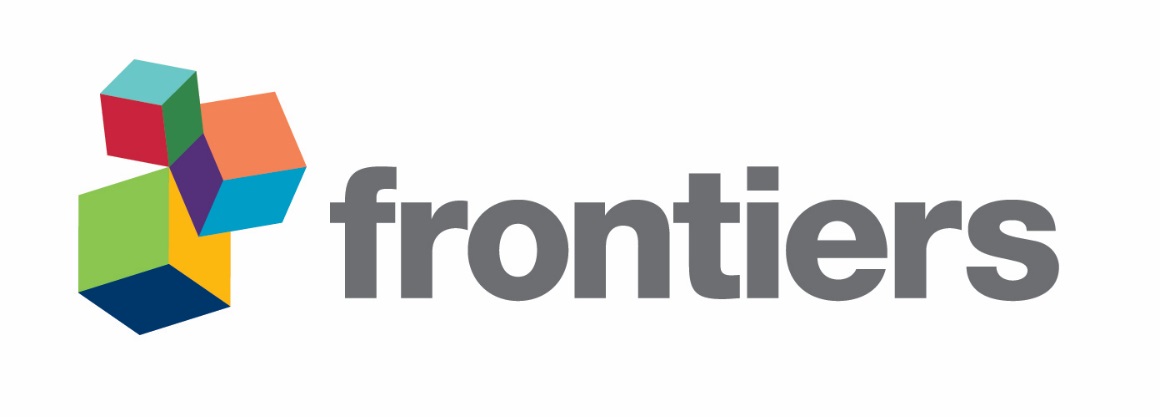
**
